# A Randomized Controlled Trial Studying the Effect of Medication Adherence Tools in Patients With Heart Failure

**DOI:** 10.7759/cureus.93238

**Published:** 2025-09-25

**Authors:** Bhupendra Pawar, Pamila Dua, Sandeep Seth, Subir K Maulik, KH Reeta

**Affiliations:** 1 Pharmacology, All India Institute of Medical Sciences, New Delhi, New Delhi, IND; 2 Cardiology, All India Institute of Medical Sciences, New Delhi, New Delhi, IND

**Keywords:** drug compliance, heart failure, medication adherence, morisky-green-levine (mgl) scale, randomized controlled trial

## Abstract

Background

Heart failure significantly contributes to the global increase in morbidity and mortality. In managing such chronic conditions, medication adherence plays a critical role in patient outcomes. Given the extensive consequences associated with non-adherence in heart failure, it is crucial to implement focused interventions that support and enhance compliance of patients in taking their prescribed medications. Therefore, this study aims to determine the effect of medication adherence tools (MAT) as an intervention to improve medication adherence and functional capacity in patients with heart failure.

Methods

In a prospective, open-label, randomized controlled study, 200 patients with heart failure were recruited, with 100 in the control group who received the standard prescribed drugs and 100 in the intervention group who received the intervention in addition to the standard prescribed drugs. A multidrug ABCD drug regimen was used for better understanding and distribution of medicines The four classes of drugs included (A) angiotensin-converting enzyme inhibitors/angiotensin II receptor blockers/angiotensin receptor neprilysin inhibitor, (B) beta-blockers/funny channel blocker (ivabradine), (C) companion drugs (digoxin, hydralazine+isosorbide dinitrate, intravenous iron, statins, oral anticoagulants/antiplatelets, antiarrhythmics, etc.), and (D) diuretics/mineralocorticoid receptor antagonists. The intervention included the ABCD drug regimen as a pharmacological approach, health education, and MAT, such as the Hriday Card, Dhadkan 2 mobile application, and labeled separate zip locks for dispensing the drugs separately as per the ABCD drug regimen. Medication adherence was assessed through the Morisky-Green-Levine (MGL) adherence scale, and quality of life assessment was done using a Likert scale. Further, physical ability was assessed by the capacity to climb floors, ability to do routine household work, and capability to walk 500 m without taking a rest.

Results

Based on the MGL adherence scale, 42 (21%) patients showed high, 132 (66%) showed medium, and 26 (13%) showed low adherence at baseline, including both the study groups. The reasons for non-adherence were found to be poor memory in 48 (24%), travelling outstation in 42 (21%), ran out of medicines in 32 (16%), adverse effects of medicines in 26 (13%), felt worse while taking medicine in 14 (7%), felt better so stopped taking medicine in 14 (7%), less affordability in six (3%), and other than these reasons in 18 (9%) of the patients. Only 40 (20%) patients demonstrated non-adherence to at least one specific class of medication, with non-adherence being maximum with diuretics. Significant improvement in medication adherence was observed in the intervention group in terms of improvement in the MGL score. There was also a significant improvement in the quality of life and the physical ability in the patients of the intervention group.

Conclusion

The findings indicate a significant occurrence of medication non-adherence among patients with heart failure within the studied population. Implementing a comprehensive, integrated intervention strategy that includes MAT led to enhanced adherence to the prescribed medications, along with notable improvements in patients’ quality of life and physical functioning. Consequently, incorporating MAT as an adjunctive measure could be recommended as an additional step toward improved outcomes in the management of patients with heart failure.

## Introduction

Heart failure is one of the most prevalent cardiovascular health issues in India and globally. It is a leading contributor to rising morbidity, mortality, reduced quality of life, and increased treatment cost among the affected patients [1,2]. There are multiple causes and factors leading toward non-adherence to medicines when we specifically talk about cardiovascular diseases, as their management requires long-term medication to slow down the progression of the disease [3].

Medication adherence is a key predictor of hospitalization and mortality in individuals with heart failure [4]. Poor adherence is linked to worse outcomes, while improved adherence can enhance the quality of life and reduce cardiovascular events [5]. However, adherence is influenced by social determinants of health, including financial barriers, health literacy, and access to care. These factors are increasingly recognized as critical in cardiology, shaping disparities in treatment and outcomes [6]. Therefore, early and long-term interventions to improve medication adherence become crucial [7]. A lot of clinical trials have employed different methods for improving medication adherence, such as educating patients, medicine reminding techniques, behavioral changes, and systematized medication regimes [8]. The different types of interventions to improve medication adherence can be categorized into patient-oriented, healthcare provider-oriented, and system-oriented based on their target [9].

A study conducted in an Indian setup utilizing digital therapeutics employing mHealth, telehealth, smart devices, sensors, wearables, health information technology, and personalized medicine showed improvement in medication adherence in patients with heart failure [10]. A study conducted on patients at six South Australian investigator centers reported significant improvement in medication adherence using a dose reminder application as an intervention [11]. Employing the use of tailored text messaging and pill box organizers in patients with heart failure at a tertiary care hospital in Iran showed improved medication adherence [12]. A study conducted in a tertiary care hospital in London demonstrated improvement in medication adherence where specialized heart failure pharmacists were employed to help in drug prescription clinics [13].

A comprehensive systematic review and meta-analysis of randomized trials found that multifaceted interventions such as patient education, reminder systems, self-care support, and provider- or technology-based strategies significantly improved medication adherence and the quality of life and reduced hospital readmissions and mortality in patients with heart failure [14]. It is important to focus on long-term outcomes of these interventions, addressing the heterogeneity in study designs and adherence measurement methods. Therefore, the present study was planned to assess the effect of medication adherence tools (MAT) in patients with heart failure in an Indian tertiary care hospital.

## Materials and methods

Study setting and study design

This was a prospective, open-label, randomized controlled, parallel-arm trial conducted at the All India Institute of Medical Sciences (AIIMS), New Delhi, India, between September 2018 and January 2020. The Institutional Ethics Committee of AIIMS, New Delhi, issued approval IECPG-338/18.07.2018/RT-17/30.08.2018. The trial was registered prospectively with the Clinical Trials Registry of India (CTRI) under registration number CTRI/2018/10/015956. Patients attending the Cardiology Outpatient Department (OPD) were recruited.

Eligibility criteria

Patients aged 18-60 years with New York Heart Association (NYHA) class 2 or 3 heart failure of either gender visiting the Cardiology OPD were included in the study. The participants already recruited for other trials, the participants with any major organ dysfunctions such as renal dysfunction (blood urea>50 mg% and serum creatinine>1.8 mg%) and hepatic dysfunction (aspartate aminotransferase/alanine aminotransferase/alkaline phosphatase/total serum bilirubin>3 times the upper normal limit), and pregnant and lactating women were excluded. Further, the participants with rapidly progressing disease and with other comorbid conditions such as uncontrolled diabetes mellitus, uncontrolled hypertension, and malignancy were also excluded. All the data were documented in a case record form.

Intervention

The participants were randomly allocated into two groups. The control group (n=100) received the standard prescribed medications, and the intervention group (n=100) received the intervention in addition to the standard prescribed medications. The intervention included the ABCD drug regimen as a pharmacological approach, health education, and MAT, such as the Hriday Card, Dhadkan 2 application, and labeled separate zip locks for dispensing the medications separately as per the ABCD drug regimen.

The multidrug ABCD drug regimen was used for a better understanding and distribution of medicines. The four classes of drugs included (A) angiotensin-converting enzyme inhibitors/angiotensin II receptor blockers/angiotensin receptor neprilysin inhibitor, (B) beta-blockers/funny channel blocker (ivabradine), (C) companion drugs (digoxin, hydralazine+isosorbide dinitrate, intravenous iron, statins, oral anticoagulants/antiplatelets, antiarrhythmics, etc.), and (D) diuretics/mineralocorticoid receptor antagonists.

Health education was used as a non-pharmacological approach, which included patient counseling and education, lifestyle changes (e.g., advice on contraception, smoking, and alcohol), immunization and antibiotic prophylaxis, diet and nutrition (salt restriction and fluid intake), exercise training, and rehabilitation.

MAT comprised the following: The first is the Hriday Card. This is a four-page tool developed to promote medication adherence and enhance patient care in the management of heart failure. Page 1 included essential information about the patient and their primary care provider. Page 2 outlined the patient’s diagnosis, prescribed medications, and vaccination records. Page 3 was dedicated to daily symptom tracking, with a color-coded system (red, yellow, and green zones) providing guidance on when to seek medical attention. Page 4 offered lifestyle modification tips to support heart failure management. In addition, a supplementary sheet was provided for daily weight monitoring, along with a medicine chart detailing all prescribed medications, including their dosages and frequency [15]. Figures are given in the Appendices.

**Figure 1:**
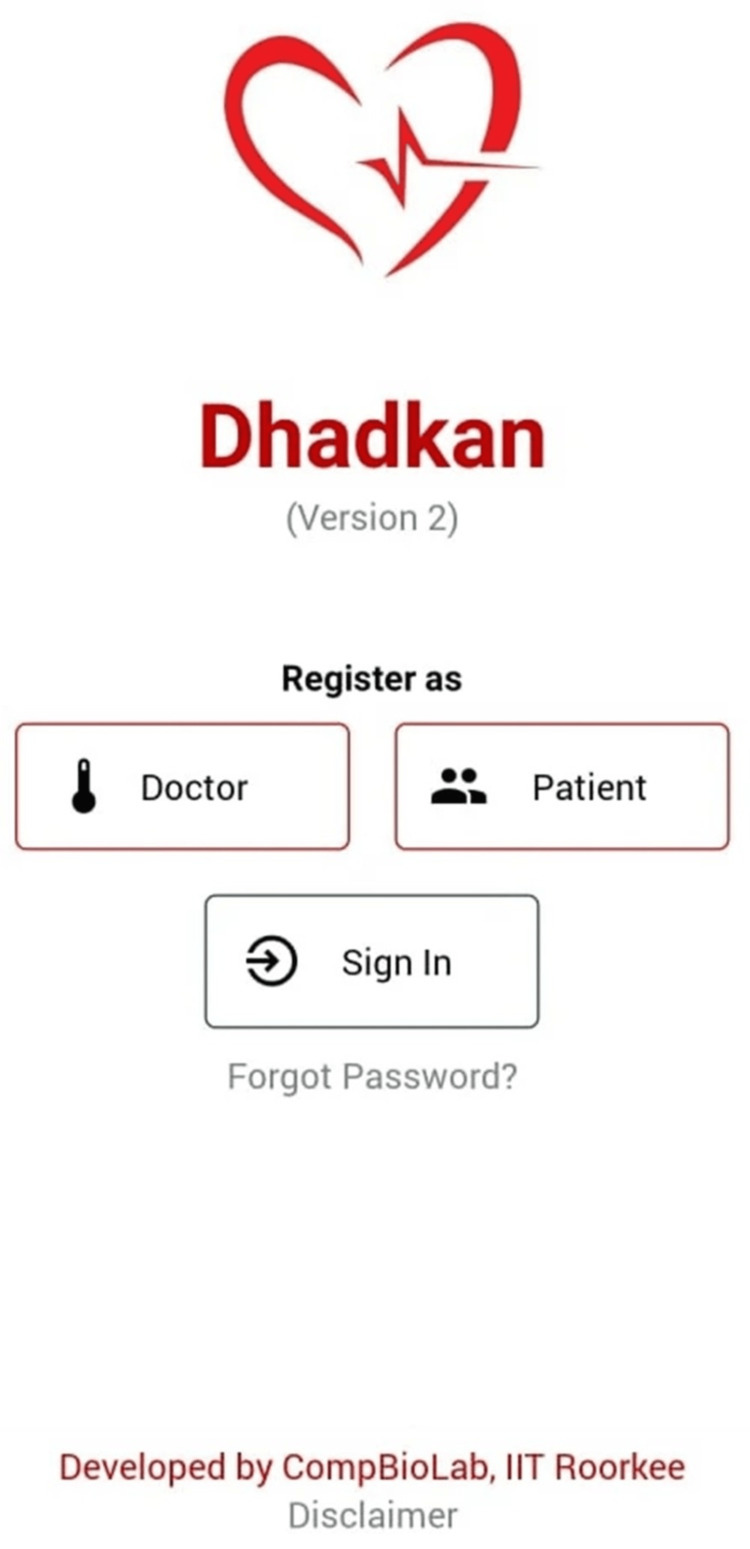
Dhadkan 2 application

The second is the Dhadkan 2 application (Figure 1). This mobile application enabled the patients to submit their weekly blood pressure, heart rate, and weight readings to the registered healthcare professionals, including doctors, nurses, and paramedics. Patients could also upload photographs of their ECG results and medications, facilitating effective communication between them and their care providers. The application automatically generated alerts for both patients and healthcare professionals if significant changes in the submitted data suggested a possible onset of heart failure [16].

The third is the ABCD regimen prescription with separate zip locks for each class. Prescriptions were provided in the ABCD format, and the patients were instructed to store their medications in individually labeled zip-lock bags for each class of medications.

All participants were assessed at baseline and at 12±2-week follow-up.

Measurement of adherence

The Morisky-Green-Levine (MGL) adherence scale was used to assess medication adherence among the participants via a publicly available four-question questionnaire [17]. The developer, Dr. Donald E. Morisky, was notified of its use in this study. Each question addresses the patient’s medication-taking behavior and is answered with “yes” (scored 1) or “no” (scored 0). The total score, calculated by summing the responses, categorizes adherence as high (0), medium (1-2), or low (3-4). The questions were as follows: (1) Do you ever forget to take your medicine? (2) Are you careless about taking your medicine? (3) Do you stop taking medicine when you feel better? (4) Do you stop when you feel worse after taking it?

Quality of life assessment

**Figure 2:**
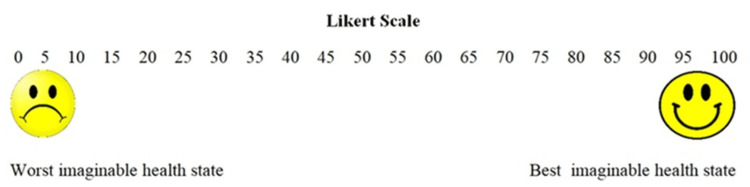
Likert scale

Quality of life was measured using a Likert scale ranging from 0 to 100 in increments of 5, where 0 represented the worst imaginable health state and 100 the best [18]. After explaining the scale, patients rated their own health, with higher scores indicating better health outcomes (Figure 2).

Additionally, physical capacity was assessed through several parameters: the number of floors a patient could climb (with inability to climb even one floor being scored as 0), ability to perform daily household tasks (scored 1 for capable and 0 for not), and walking ability (scored 1 if able to walk 500 m without rest and otherwise 0) to evaluate functional capacity in patients with heart failure.

Permission to use the adherence scale and tools

The MGL questionnaire, Likert scale, Hriday Card, and Dhadkan 2 application used were not copyrighted and were freely available in the public domain.

Randomization and allocation concealment

The participants were randomized in a 1:1 ratio to either the intervention or control group using block randomization with a fixed block size of 4. The randomization sequence was generated by an independent statistician using a computer-generated list to ensure equal group sizes and reduce allocation bias.

Allocation concealment was maintained through the use of sequentially numbered, opaque, sealed envelopes (SNOSE), which were prepared and stored by a third party not involved in recruitment or assessment. Envelopes were opened only after participant consent was obtained and eligibility confirmed.

Blinding

Due to the nature of the intervention (the use of tools, applications, and educational materials), this was an open-label trial. However, data entry personnel and statisticians were blinded to group allocation during data analysis to minimize bias.

Outcomes

The primary outcome measure is improvement in medication adherence by measuring the change in the Morisky-Green-Levine (MGL) adherence scale score.

The secondary outcome measures are (a) change in the quality of life, assessed using a Likert scale, and (b) change in functional capacity, measured through the number of floors climbed, ability to perform household tasks, and ability to walk 500 m without rest.

Recruitment and follow-up

Recruitment began in September 2018 and was completed by December 2019. Follow-up assessments were conducted at 12±2 weeks after baseline.

**Figure 3:**
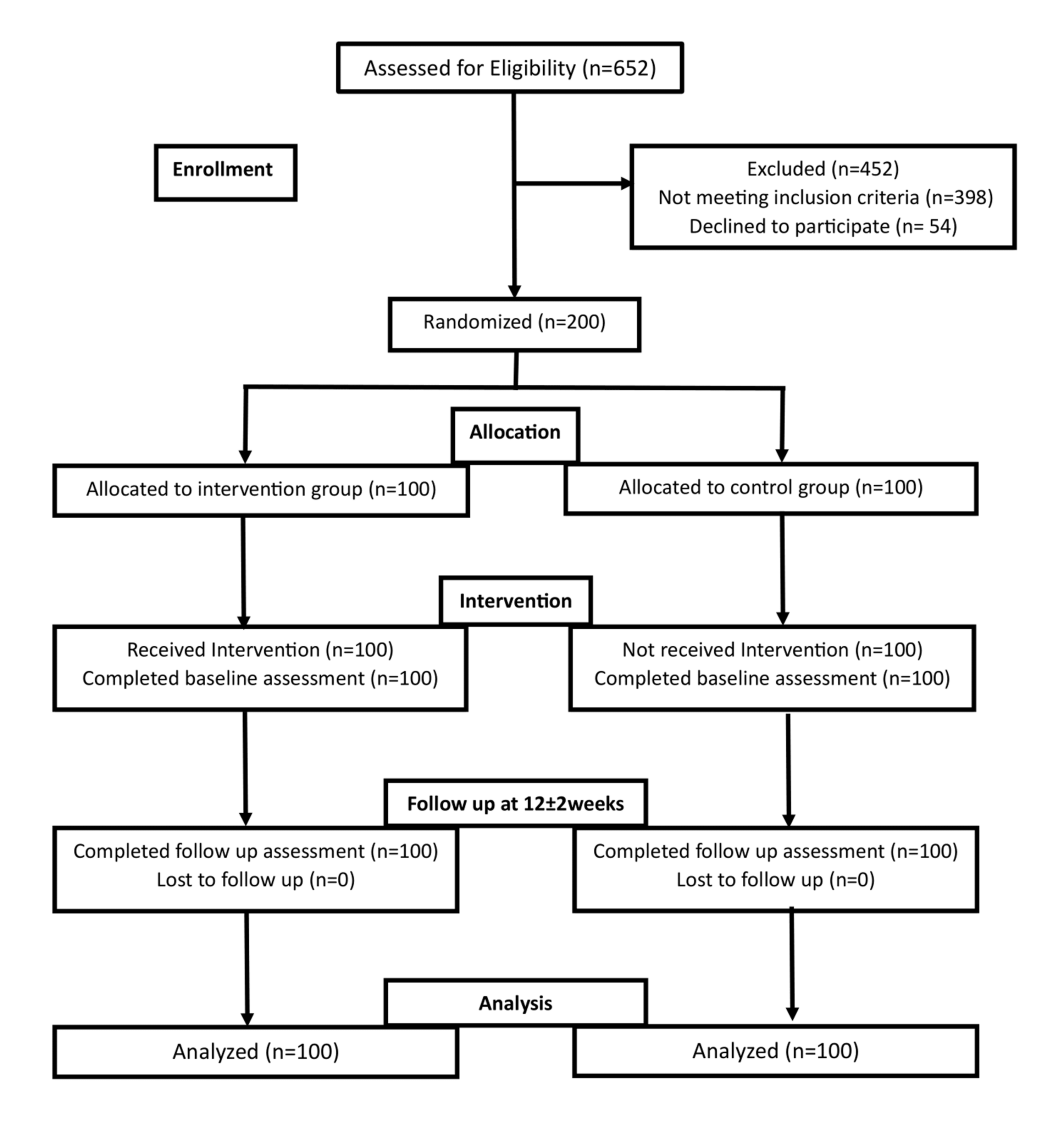
CONSORT flow diagram CONSORT: Consolidated Standards of Reporting Trials

A Consolidated Standards of Reporting Trials (CONSORT) flow diagram (Figure 3) details the number of participants enrolled, allocated, lost to follow-up, and analyzed in each group.

Sample size calculation

To the best of our knowledge, this study is the first of its kind to utilize MAT as an interventional tool to assess the effect of medication adherence in patients with heart failure. Prior to the main study, a pilot assessment was conducted to determine the rate of non-adherence among a cohort similar to our study population. Non-adherence was found to be approximately 50%, which was consistent with findings from an earlier study [19]. Based on this estimate, the sample size was calculated assuming a 50% prevalence of poor adherence, with a 95% confidence level (α=0.05) and a 10% margin of error. Accordingly, the final study included two groups: an intervention group (n=100), which received the standard prescribed drugs with the intervention, and a control group (n=100), which received only the standard prescribed drugs.

Statistical analysis

Statistical analysis was conducted in collaboration with the Department of Biostatistics, AIIMS, New Delhi, using Stata software version 15 (StataCorp LLC, College Station, TX). No interim analyses were planned or conducted for this study. Categorical variables were presented as frequencies and percentages, while quantitative data were expressed as mean±standard deviation (SD). The normality of continuous data was assessed using the Shapiro-Wilk test. The data were found to be approximately normally distributed at baseline. An independent sample t-test was used to compare the means of continuous variables between the intervention and control groups. Pearson’s chi-square test was used to compare categorical outcomes between the two groups. A p-value of <0.05 was considered statistically significant.

## Results

A total of 652 patients were screened. On the basis of inclusion and exclusion criteria, a total of 200 patients diagnosed with heart failure were recruited for the study. Block randomization (blocks of four) was used to randomize them into two groups, the intervention group and the control group, with 100 patients in each group. There was no significant difference in any of their demographic parameters (Table 1).

**Table 1:** Comparison of demographic characteristics of the patients with heart failure in the intervention group and the control group at baseline *Mean±standard deviation ^#^Percentage

	Intervention group (n=100)	Control group (n=100)	P-value
Age (years)	45.12±15.56*	46.01±6.36*	0.61
Gender (male/female)	67:33^#^	61:39^#^	0.10
Weight (kg)	61.96±13.44*	61.53±10.6*	0.82
Marital status (married/unmarried)	83:17^#^	95:5^#^	-

The different causes of non-adherence in the 200 study participants at baseline are shown in Table 2.

**Table 2:** Percentage distribution of different causes of non-adherence in patients with heart failure Others include fasting, irritated, careless, drank alcohol, slept early, lazy, in haste, and in anger

Cause of non-adherence	Percentage	Frequency
Poor memory	24	48
Travelling outstation	21	42
Ran out of medicines	16	32
Adverse effects of medicines	13	26
Others	9	18
Felt worse while taking medicine	7	14
Felt better while taking medicine	7	14
Less affordability	3	6

**Figure 4:**
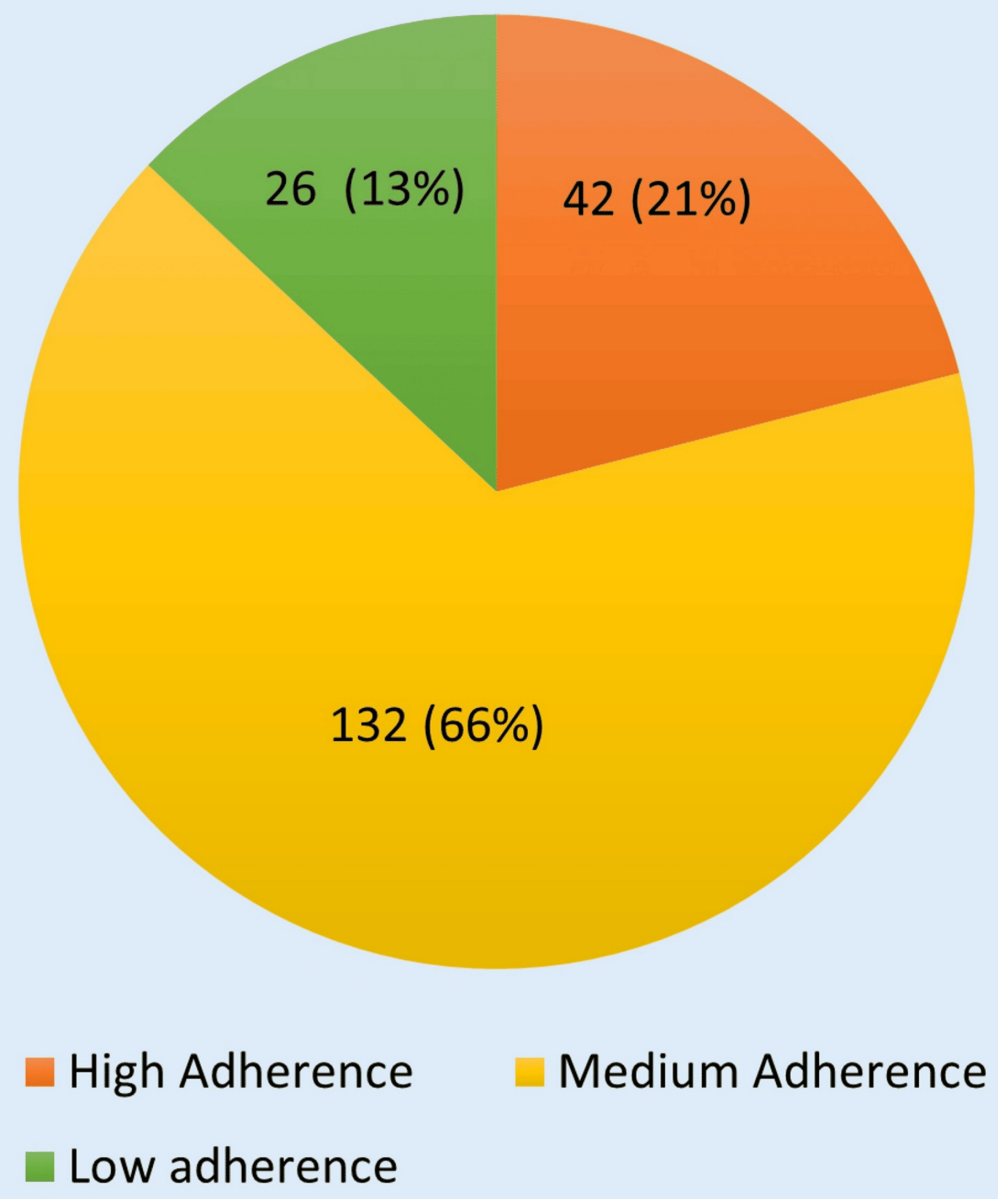
Percentage distribution of adherence in patients with heart failure Adherence of 200 patients at baseline

Only 40 (20%) patients demonstrated non-adherence to at least one specific class of medication, out of which non-adherence was maximum with diuretics. MGL score was calculated using the MGL adherence scale. Overall, 42 (21%) patients showed high adherence, 132 (66%) patients showed medium adherence, and 26 (13%) patients showed low adherence at baseline (Figure 4).

**Figure 5:**
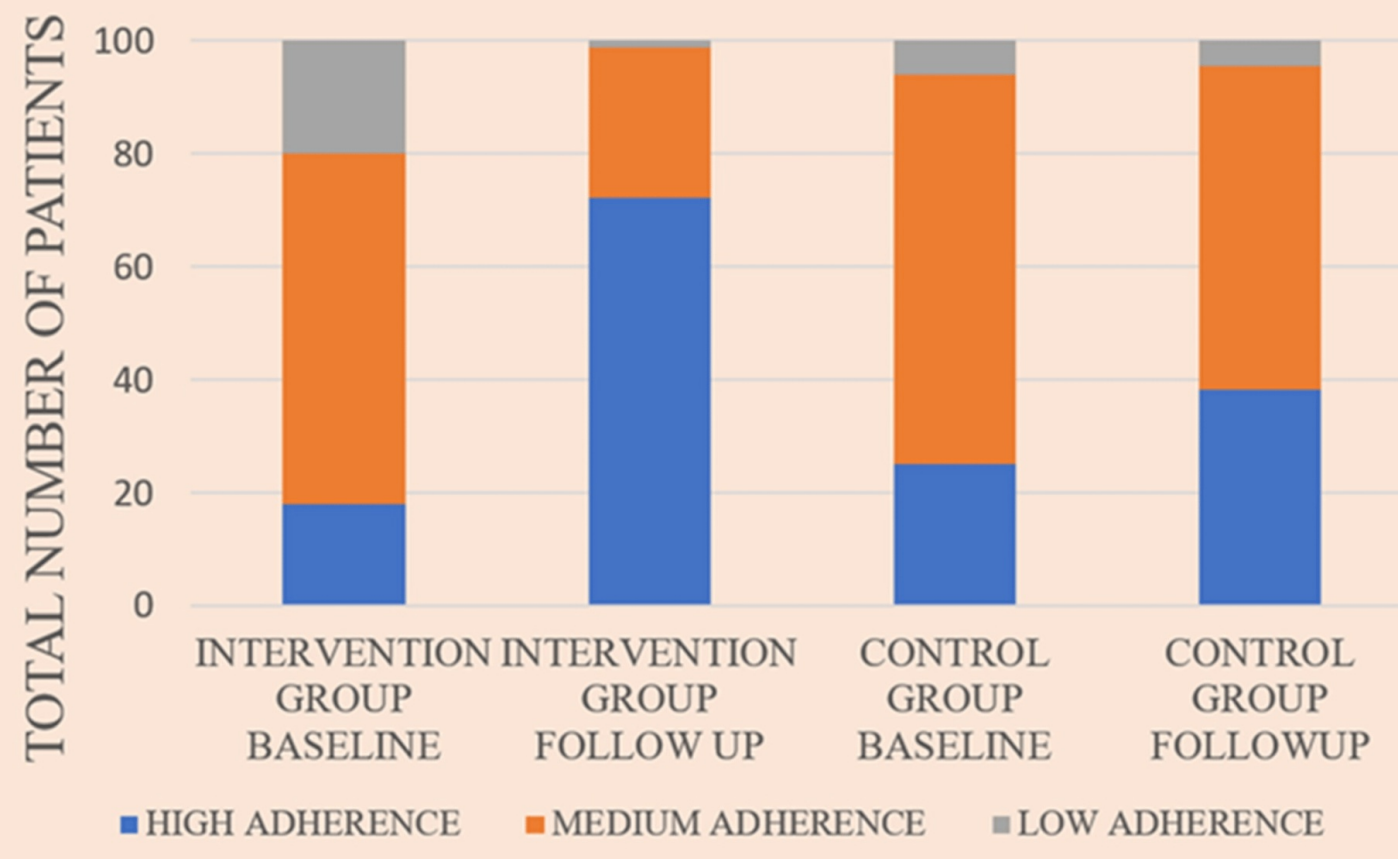
Distribution of adherence in the intervention group and the control group at baseline and follow-up

Percentage change from baseline to follow-up in types of adherence in each group is depicted in Figure 5.

The intervention group showed a significant improvement in overall MGL adherence scores compared to the control group (Table 3).

**Table 3:** Change in MGL scores in the intervention and control groups Pearson’s chi-square(1)=32.7922; p<0.0001 MGL: Morisky-Green-Levine

MGL score	Intervention group (n=100)	Control group (n=100)	Total (n=200)
Increased or no change	23 (23.0%)	65 (65.0%)	88 (44.0%)
Decreased	77 (74.0%)	35 (35.0%)	112 (56.0%)
Total	100 (100%)	100 (100%)	200 (100%)

Quality of life assessed using the Likert scale was also significantly better in the intervention group (Table 4).

**Table 4:** Change in Likert scores in the intervention and control groups Pearson’s chi-square(1)=5.4912; p=0.01

Likert score	Intervention group (n=100)	Control group (n=100)	Total (n=200)
Decreased or no change	29 (29.0%)	45 (45.0%)	74 (37.0%)
Increased	71 (71.0%)	55 (55.0%)	126 (63.0%)
Total	100 (100%)	100 (100%)	200 (100%)

Additionally, physical ability, measured by the capacity to climb floors (Table 5), perform household work (Table 6), and walk for 500 m (Table 7), showed notable improvement in the intervention group compared to the control group.

**Table 5:** Change in capacity to climb floors in the intervention and control groups Pearson’s chi-square(1)=8.68; p=0.0032

Climb floors	Intervention group (n=100)	Control group (n=100)	Total (n=200)
No improvement	61 (61.0%)	80 (80.0%)	141 (70.5%)
Improvement	39 (39.0%)	20 (20.0%)	59 (29.5)
Total	100 (100%)	100 (100%)	200 (100%)

**Table 6:** Change in capacity to do household work in the intervention and control groups Pearson’s chi-square(1)=8.14; p=0.0043

Household work	Intervention group (n=100)	Control group (n=100)	Total (n=200)
No improvement	79 (79.0%)	93 (93.0%)	172 (86%)
Improvement	21 (21.0%)	7 (7.0%)	28 (14%)
Total	100 (100%)	100 (100%)	200 (100%)

**Table 7:** Change in the ability to walk in the intervention and control groups Pearson’s chi-square(1)=4.31; p=0.038

Walking activity	Intervention group (n=100)	Control group (n=100)	Total (n=200)
No improvement	85 (85.0%)	94 (94.0%)	179 (89.5%)
Improvement	15 (15.0%)	6 (6.0%)	21 (10.5%)
Total	100 (100%)	100 (100%)	200 (100%)

No harms or unintended effects were reported in either group.

## Discussion

In the present study, the adherence category assessed in patients with heart failure was classified as high, medium, and low based on the MGL adherence scale. It offers several advantages, including ease and speed of administration due to its small scale, and it is effective in identifying the causes of non-adherence. It has been validated in many diseases and is the most adaptable scale across different populations [17]. Therefore, this scale is widely used in research.

The Change the Management of Patients with Heart Failure (CHAMP-HF) study conducted in the United States in outpatients with heart failure with reduced ejection fraction using MGL score measurement showed 31.7% non-adherence to the medications [20]. These results align with broader literature that consistently indicates high rates of non-adherence among patients with heart failure. Variability across studies may stem from differences in health system infrastructure, access to medication, health literacy, and cultural attitudes toward chronic disease management. A study on patients with heart failure visiting Debre Berhan Comprehensive Specialized Hospital (DBCSH) referral clinics in Ethiopia using the MGL scale showed a significant positive correlation between treatment satisfaction and medication adherence [21]. A pharmacist intervention was used in similar patients visiting DBCSH as a mode to improve medication adherence, where the pre-intervention phase showed 39.1% of patients with low adherence, 54.6% with medium medication adherence, and 6.3% with high adherence and the post-intervention phase showed 1.7% of patients with low adherence, 61.9%% with medium medication adherence, and 36.4% with high adherence [22].

A study conducted on patients with heart failure in a hospital in Indonesia showed significant improvement in medication adherence measured by the Morisky Medication Adherence Scale-8 (MMAS-8), where a tele-motivational interviewing approach was used as an effective intervention [23]. A study conducted in a tertiary care hospital in the rural population of India showed 28.37% high adherence in patients with heart failure when assessed by the Morisky Medication Adherence Scale-4 (MMAS-4) [24]. A study conducted in a tertiary care center in Thailand in patients with chronic heart failure reported 38.3% high adherence, 50.0% medium adherence, and 11.7% low adherence, where MMAS-8 was used for assessment [25]. In the present study, we found that only 21% patients showed high adherence, whereas 66% of patients were of medium adherence, and 13% of patients were of low adherence, which is in concordance with previous studies showing the high prevalence of non-adherence in patients with heart failure. A study conducted on patients with heart failure in Brazil showed self-care, family income, depression, and alcohol consumption as factors that contributed to low medication adherence [26]. In our study, an attempt was made to identify the reasons for non-adherence in our patients with heart failure. The reasons were poor memory in 23.81% of patients, travelling outside in 21.03% of patients, ran out of the medicines in 15.48% of patients, adverse effects of medicines in 13.10% of patients, felt worse while taking medicine in about 7.14% of patients, felt better so stopped taking medicine in 6.75% of patients, less affordability in 3.17% of patients, and other reasons in 9.52% of patients.

On assessment based on different categories, the diuretic class of drugs had the maximum non-adherence (76%) out of all the classes of drugs used in patients with heart failure. The increased frequency of urination, along with disruptions to daytime activities and nighttime sleep, was the primary reason for non-adherence to diuretics. On the assessment of adherence toward individual drugs in each category, non-adherence was not related to any specific drug in 80% of all patients.

The present study showed a high prevalence of non-adherence to medicines in patients with heart failure in the Indian population, supporting the already existing data [1]. The mean age and weight of the patients in the study were 45.6 years and 61.7 kg, respectively; 36% were women, and 64% were men. Our findings were similar to the findings of an earlier study conducted in India in patients with heart failure with reduced ejection fraction, which reported a mean age of 56 years and a 23% female population [27].

We have used an integrated approach to improve medication adherence and functional capacity in patients with heart failure, which included a multidrug ABCD drug regimen as a pharmacological approach, health education, and MAT, such as the Hriday Card, Dhadkan 2 application, and labeled separate zip locks for every class of drug. A systematic review on medication adherence interventions for older adults with heart failure showed that the application of combined approaches of educational, behavioral, and affective interventions with a focus on medication adherence demonstrated improved outcomes [28].

In our study, we have used a four-class ABCD drug regimen as a pharmacological approach for a better understanding and distribution of the medicines to help both the prescribing physician and the patient. The Hriday Card gave basic information about the patient, diagnosis, details about daily symptoms to be monitored, when to seek medical help, and different lifestyle modification measures to control heart failure. The Dhadkan 2 application was a mobile application where the patient needed to send his blood pressure, heart rate, and weight to the registered medical personnel (doctor/nurse/paramedic) once a week, with a facility to send photographs (ECG and medicines). This application acted as a medium for communication between the healthcare provider and the patient.

Separate labeled zip locks for each class of medications were provided at the first visit, and they were asked to bring them to each OPD visit, where it was checked and corrected and explained to the patient if required. To the best of our knowledge, all these interventions are new and are being used for the first time in the present study.

In our study, multiple interventions used as an add-on therapy with standard prescribed drugs showed significant improvement in medication adherence in terms of improvement in the MGL score. The present study is the first of its kind to study the effect of medication adherence tools in patients with heart failure. A meta-analysis study of randomized controlled trials using a broad range of interventions showed improvement in 10% of patients with heart failure [14]. A study conducted on patients with chronic heart failure with poor adherence showed improvement in medication adherence when the medication was provided by the nurse [29]. The two groups were also compared to assess the functional capacity of the patients with heart failure, which showed a significant improvement measured by improvement in the Likert score. Significant improvements in physical ability in terms of climbing floors, doing day-to-day household work, and walking capacity were also observed. An intervention study including structured telephone support and short message service text messaging as an intervention reported improvement in heart failure outcomes [30]. A narrative review study using a comprehensive search in the databases showed that educational interventions using face-to-face and teach-back training, home visit by follow-up phone call, group training, and e-learning had a significant effect on self-care behavior in patients with heart failure [31]. In this study, we observed a high level of non-adherence in patients with heart failure in a tertiary care center in India. We also identified various causes of non-adherence that need to be addressed to improve medication adherence. In our study, interventions using a combined pharmacological approach, health education, and different tools to increase medication adherence with the appropriate use of technology showed improvement in adherence level and functional capacity of the patients with heart failure. However, novel ideas need to be implemented in the interventional approach to get better outcomes.

There were certain limitations to our study. The questionnaires that we used were mostly dependent on patient recall. Therefore, there were always chances of recall bias. The patient may even hide the details in fear of unacceptance by the health worker. The patients in the control group also interacted with the investigator about their medications, and different questionnaires were being assessed. This attention can also lead to improvement in medication adherence in the control group. Not all the patients were well versed with technology, so they could not understand and utilize the technology-based intervention. Illiterate patients had difficulty understanding the educational approach of the intervention.

The findings of our study have several important clinical implications for the management of heart failure. Improved adherence through structured interventions such as the MAT approach can lead to better disease control, fewer hospitalizations, and reduced healthcare costs. The use of the ABCD regimen with zip-lock categorization offers a practical and patient-friendly method for organizing medications, enhancing the ease of use, and compliance. Additionally, mobile health tools such as the Dhadkan 2 application support real-time monitoring and proactive management, facilitating timely interventions. Integrating these components into routine outpatient care may strengthen chronic disease management programs, offering a scalable and low-cost model that can be embedded into standard care protocols not only for heart failure but also for other chronic conditions.

## Conclusions

This study revealed a high rate of medication non-adherence among patients with heart failure within our population. It demonstrated that the implementation of a comprehensive, integrated intervention, specifically tailored with medication adherence tools, significantly improved adherence levels. The present research not only assessed adherence but also linked these improvements to meaningful enhancements in patients’ quality of life and physical functioning.

## Figures and Tables

**Figure 6:**
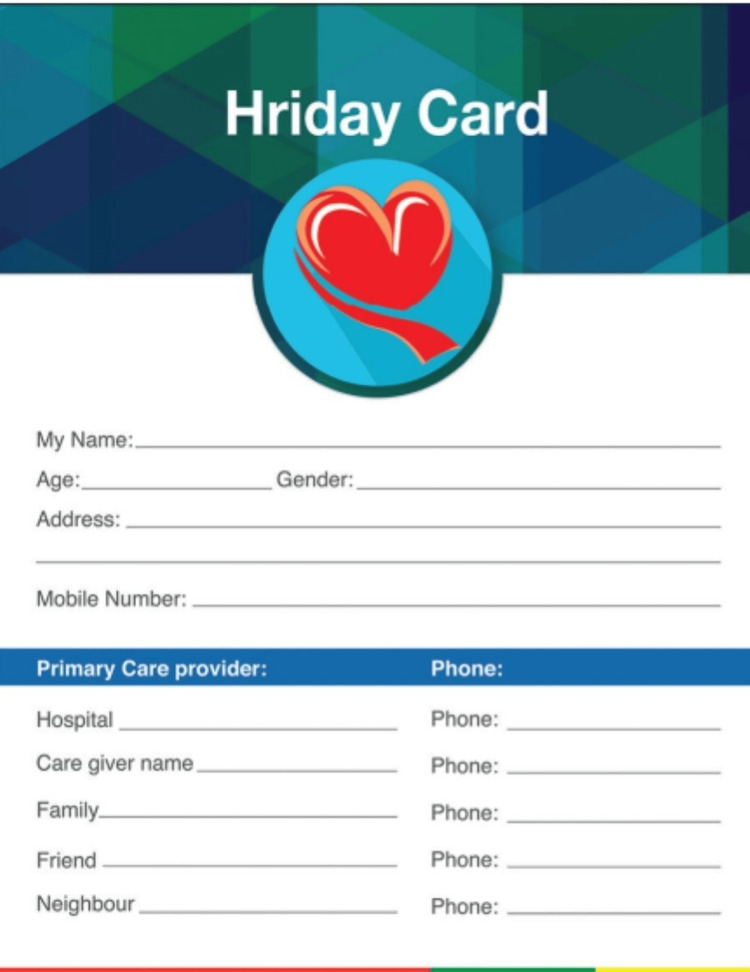
Page 1 of the Hriday Card Image from Seth and Vashista [15]. Licensed under Creative Commons Attribution (CC BY) (https://s100.copyright.com/AppDispatchServlet?publisherName=medknow&publication=JPCS&title=The%20Hriday%20Card:%20A%20Checklist%20for%20Heart%20Failure&publicationDate=01012017&author=Sandeep%20Seth%20and%20Shivani%20Vashista&volumeNum=3&issueNum=1&copyright=%C2%A9%202017%20Journal%20of%20the%20Practice%20of%20Cardiovascular%20Sciences%20%7C%20Published%20by%20Wolters%20Kluwer%20%E2%80%93%20Medknow&contentID=01881846-201703010-00003&orderBeanReset=True)

**Figure 7:**
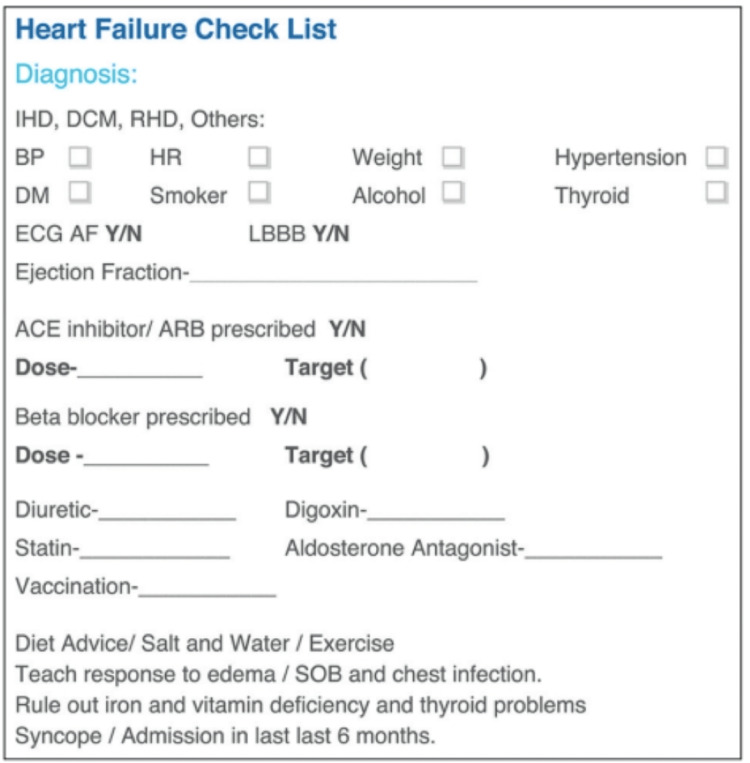
Page 2 of the Hriday Card Image from Seth and Vashista [15]. Licensed under Creative Commons Attribution (CC BY) (https://s100.copyright.com/AppDispatchServlet?publisherName=medknow&publication=JPCS&title=The%20Hriday%20Card:%20A%20Checklist%20for%20Heart%20Failure&publicationDate=01012017&author=Sandeep%20Seth%20and%20Shivani%20Vashista&volumeNum=3&issueNum=1&copyright=%C2%A9%202017%20Journal%20of%20the%20Practice%20of%20Cardiovascular%20Sciences%20%7C%20Published%20by%20Wolters%20Kluwer%20%E2%80%93%20Medknow&contentID=01881846-201703010-00003&orderBeanReset=True) IHD, ischemic heart disease; DCM, dilated cardiomyopathy; RHD, rheumatic heart disease; BP, blood pressure; DM, diabetes mellitus; HR, heart rate; AF, atrial fibrillation; Y/N, yes/no; LBBB, left bundle branch block; ACE, angiotensin-converting enzyme; ARB, angiotensin receptor blockers; SOB, shortness of breath

**Figure 8:**
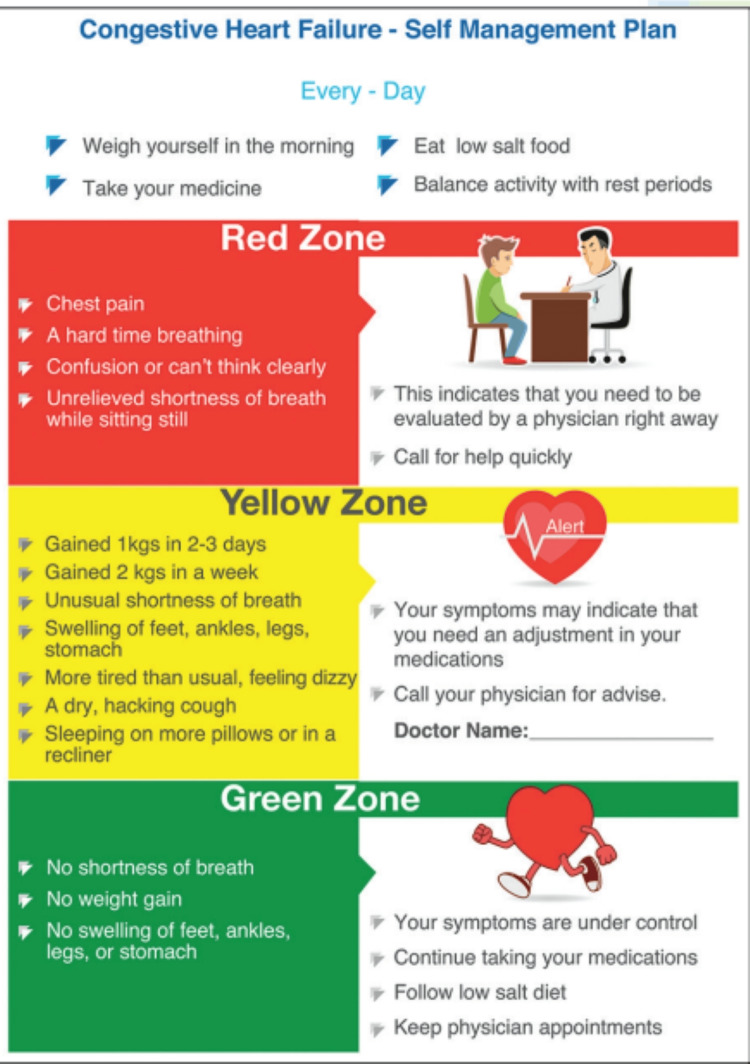
Page 3 of the Hriday Card Image from Seth and Vashista [15]. Licensed under Creative Commons Attribution (CC BY) (https://s100.copyright.com/AppDispatchServlet?publisherName=medknow&publication=JPCS&title=The%20Hriday%20Card:%20A%20Checklist%20for%20Heart%20Failure&publicationDate=01012017&author=Sandeep%20Seth%20and%20Shivani%20Vashista&volumeNum=3&issueNum=1&copyright=%C2%A9%202017%20Journal%20of%20the%20Practice%20of%20Cardiovascular%20Sciences%20%7C%20Published%20by%20Wolters%20Kluwer%20%E2%80%93%20Medknow&contentID=01881846-201703010-00003&orderBeanReset=True)

**Figure 9:**
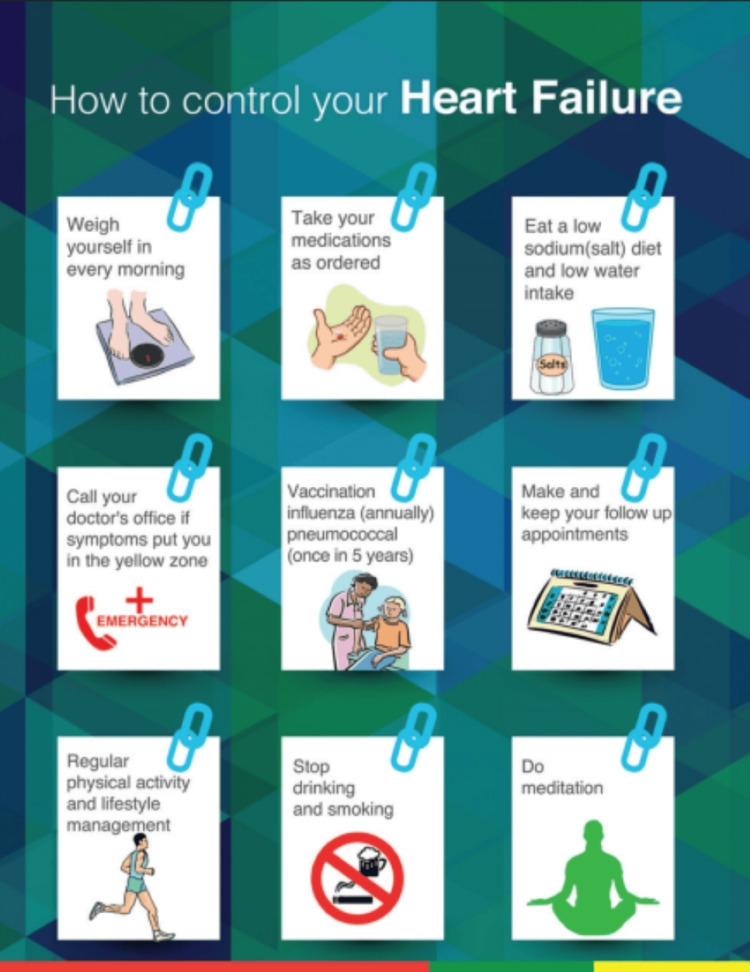
Page 4 of the Hriday Card Image from Seth and Vashista [15]. Licensed under Creative Commons Attribution (CC BY) (https://s100.copyright.com/AppDispatchServlet?publisherName=medknow&publication=JPCS&title=The%20Hriday%20Card:%20A%20Checklist%20for%20Heart%20Failure&publicationDate=01012017&author=Sandeep%20Seth%20and%20Shivani%20Vashista&volumeNum=3&issueNum=1&copyright=%C2%A9%202017%20Journal%20of%20the%20Practice%20of%20Cardiovascular%20Sciences%20%7C%20Published%20by%20Wolters%20Kluwer%20%E2%80%93%20Medknow&contentID=01881846-201703010-00003&orderBeanReset=True)

**Figure 10:**
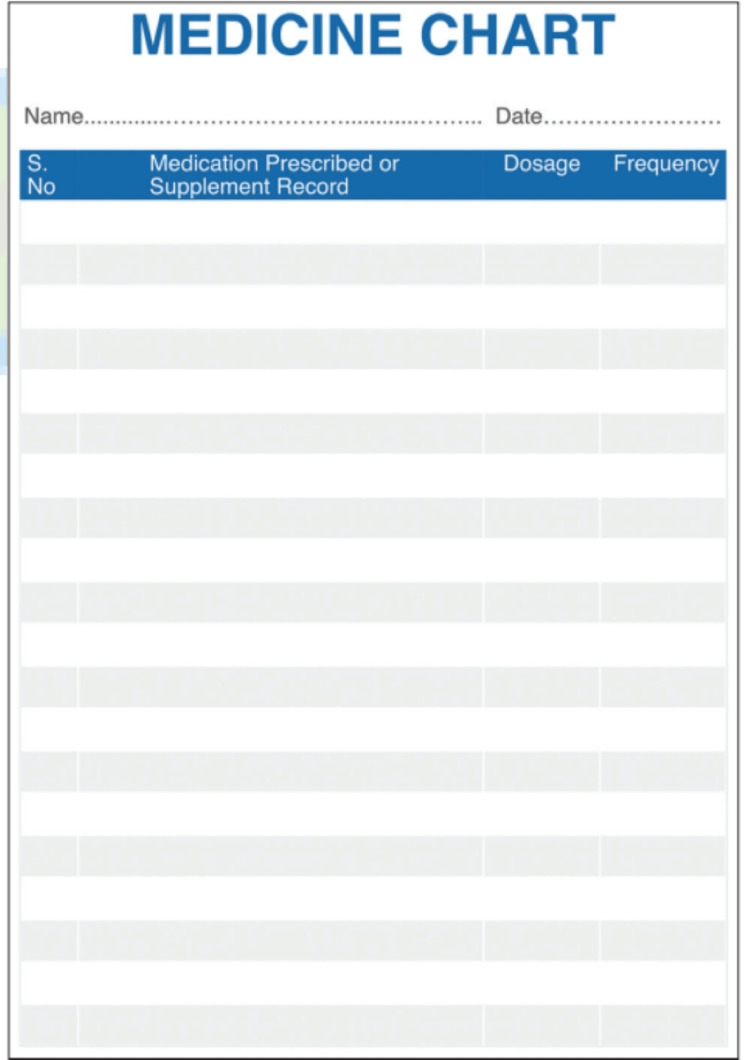
Hriday Card supplementary sheet on the front side Image from Seth and Vashista [15]. Licensed under Creative Commons Attribution (CC BY) (https://s100.copyright.com/AppDispatchServlet?publisherName=medknow&publication=JPCS&title=The%20Hriday%20Card:%20A%20Checklist%20for%20Heart%20Failure&publicationDate=01012017&author=Sandeep%20Seth%20and%20Shivani%20Vashista&volumeNum=3&issueNum=1&copyright=%C2%A9%202017%20Journal%20of%20the%20Practice%20of%20Cardiovascular%20Sciences%20%7C%20Published%20by%20Wolters%20Kluwer%20%E2%80%93%20Medknow&contentID=01881846-201703010-00003&orderBeanReset=True)

**Figure 11:**
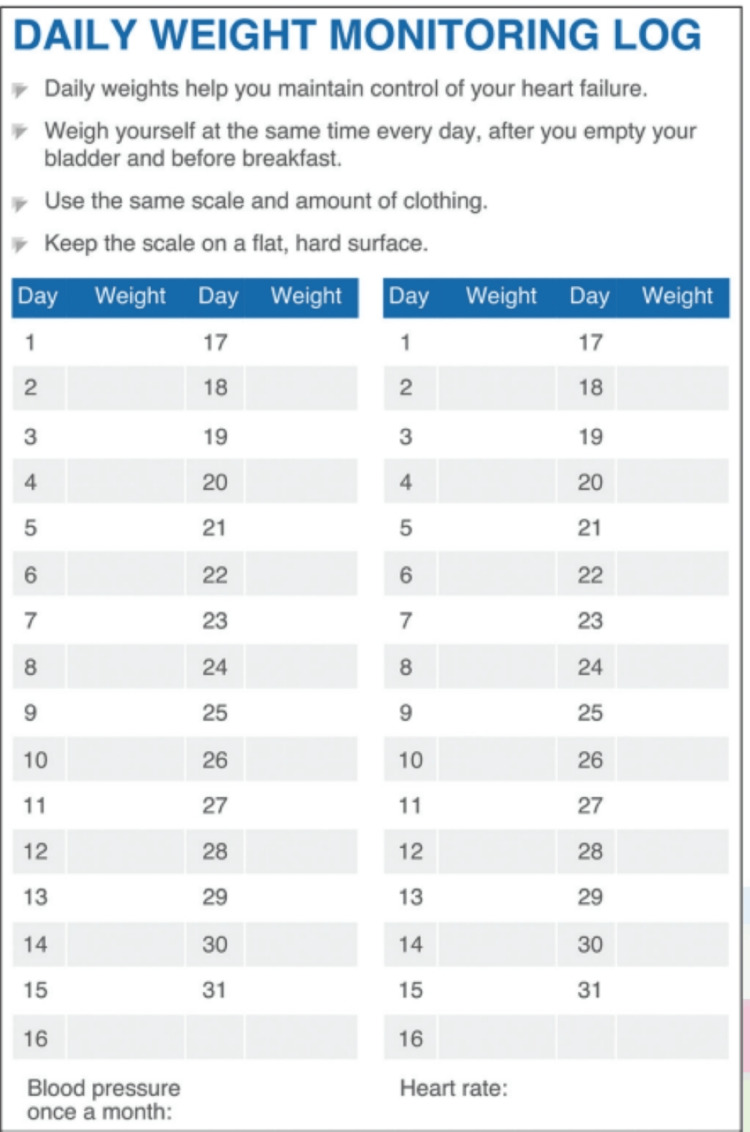
Hriday Card supplementary sheet on the back side Image from Seth and Vashista [15]. Licensed under Creative Commons Attribution (CC BY) (https://s100.copyright.com/AppDispatchServlet?publisherName=medknow&publication=JPCS&title=The%20Hriday%20Card:%20A%20Checklist%20for%20Heart%20Failure&publicationDate=01012017&author=Sandeep%20Seth%20and%20Shivani%20Vashista&volumeNum=3&issueNum=1&copyright=%C2%A9%202017%20Journal%20of%20the%20Practice%20of%20Cardiovascular%20Sciences%20%7C%20Published%20by%20Wolters%20Kluwer%20%E2%80%93%20Medknow&contentID=01881846-201703010-00003&orderBeanReset=True)

## Appendices

Hriday Card

Figures 6-11 show the content of the Hriday Card.
